# Rectal foreign body causing perforation: Case report and literature review

**DOI:** 10.1016/j.amsu.2019.10.005

**Published:** 2019-10-11

**Authors:** Youssef Shaban, Adel Elkbuli, Vasiliy Ovakimyan, Rachel Wobing, Dessy Boneva, Mark McKenney, Shaikh Hai

**Affiliations:** a Department of Surgery, Kendall Regional Medical Center, Miami, FL, USA; b University of South Florida, Tampa, FL, USA

**Keywords:** Psychiatric disease, Rectal foreign body, Anorectal trauma, Necrotizing infection, Bowel perforation

## Abstract

**Background:**

Clinicians must maintain an index of suspicion to diagnose an anorectal foreign body (FB). The patient may not be forthcoming with information secondary to embarrassment or possibly psychiatric issues. Providers must express empathy and compassion while maintaining nonjudgmental composure. Despite accounts of anal FB insertion, this pathology is lacking level one evidence-based surgical algorithms.

**Case presentation:**

A 46-year-old male psychiatric patient presented in septic shock, complaining of lower abdominal/pelvic pain starting 1 week prior. His past medical history was significant for schizophrenia, bipolar disorder, and noncompliance with medications. CT of the abdomen/pelvis revealed a rectal perforation with free air and a FB which appeared to be a screwdriver. Fluid resuscitation and broad-spectrum antibiotics were administered. In the operating room, after unsuccessful transrectal removal, an exploratory laparotomy was performed. The metallic end of the screwdriver had perforated the rectosigmoid. Resection of the perforated rectum with removal of the screwdriver, incision and drainage of a large right buttock abscess and colostomy was performed. The patient recovered and was discharged to behavioral health. At 2 weeks follow-up the patient was doing well with a functioning colostomy and reversal was planned for later this year.

**Conclusion:**

This case highlights the importance of maintaining a high index of suspicion when encountering psychiatric patients with nonspecific lower abdominal or anorectal pain with inconsistent presentations. Controversy exists regarding the type of surgical treatment in case of anorectal perforation. More research is needed to provide surgeons with evidence-based standardized methods for dealing with these rare pathologies.

## Highlights

• Maintaining a high index of suspicion when encountering patients with nonspecific lower abdominal and/or rectal pain.
• Despite the numerous reports of anal FB trauma there are no cases documenting a unique incident with a screwdriver being inserted through the anus.
• Controversy exists regarding the type of surgical treatment.

## 1 Introduction

The earliest reported cases of anorectal trauma due to foreign body (FB) insertion date back to the 1500s. Since then there have been astonishing accounts of various objects being inserted into the anus described in the literature. Objects documented include lightbulbs, sex toys, toothbrushes, drugs, cell phones, fruits, vegetables, and in one incidence a frozen pig's tail [1,2].

There have been two previously documented cases in the literature in which screwdrivers were discovered in the colon, both however, without bowel perforation. In 1861 a prisoner inserted a tool box measuring 5 × 6 inches that lead to his demise one week later. The postmortem autopsy revealed the cylindrical box in the transverse colon which contained two small saws, a steal screw, a 4-inch long gun barrel, and a screwdriver. It was later revealed that these cylindrical boxes were used quite commonly by the prisoners and were usually pushed into the anus base first. However, this prisoner had inserted the conic end first which made expulsion nearly impossible [1]. The second case, reported by Sharif et al. [3], was a 56 year-old male with a psychiatric condition who presented with left lower quadrant abdominal pain and was found to have a screwdriver inserted into the rectum on plain radiograph. Initial attempts at manual extraction were unsuccessful however they were able to eventually remove the foreign object using Kelly forceps with the patient in the jackknife position.

The most common reason, by far, for anal FB insertion is sexual pleasure, however other documented explanations include drug concealment, assault, “accidental”, psychiatric reasons, and to alleviate diarrhea or constipation [[1], [2], [3]]. The clinician must maintain an index of suspicion to accurately diagnose this condition. The patient may not be forthcoming initially with the critical information as it may be an embarrassing situation. It is fundamental that healthcare providers express empathy and compassion along with maintaining nonjudgmental composure with the highest degree of professionalism.

Despite the numerous literature accounts of surgeons’ experience with anal FB trauma, this pathology is lacking level one evidence-based standardized surgical management algorithms. This type of injury happens too infrequently for any single institution to accumulate enough cases for meaningful statistical analysis. Most of the data published are case reports, surgeon experience, and retrospective analysis of hospital specific outcomes. This work has been reported in line with the SCARE criteria [4].

## 2 Case presentation

**Fig. 1:**
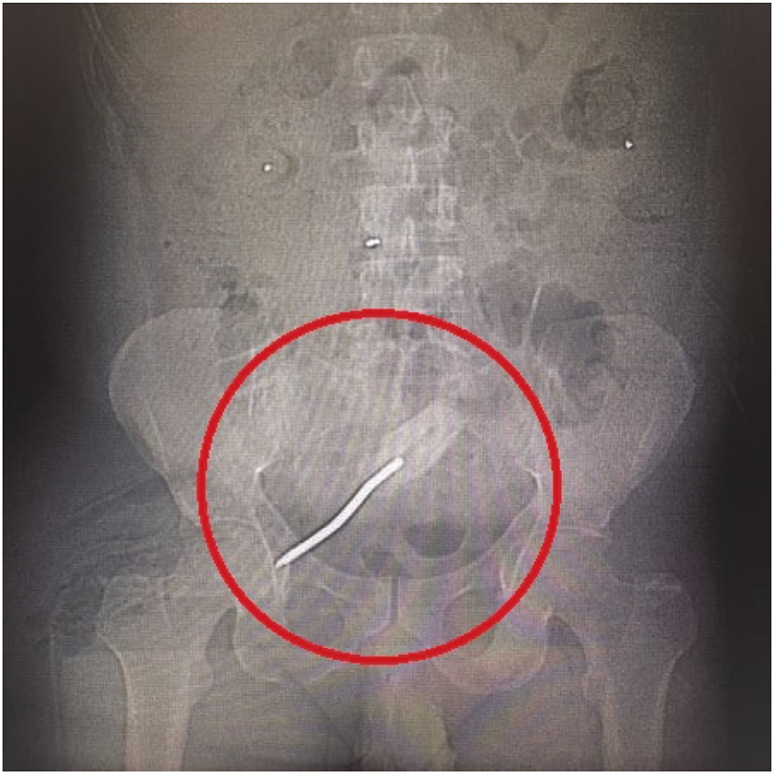
CT scout image with the foreign body identified as a screwdriver, circled in red.

**Fig. 2:**
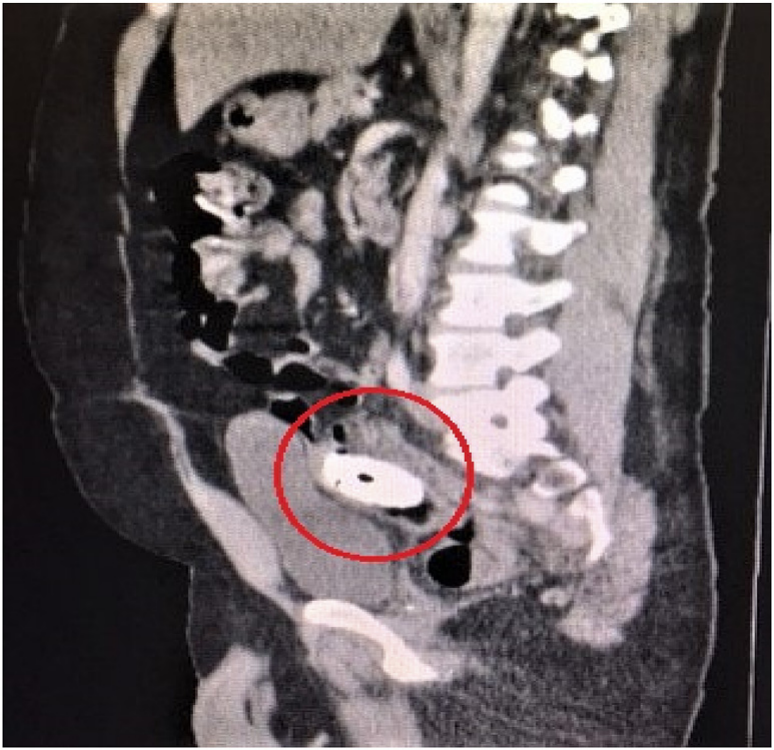
CT sagittal view image depicting the foreign body trajectory, circled in red.

**Fig. 3:**
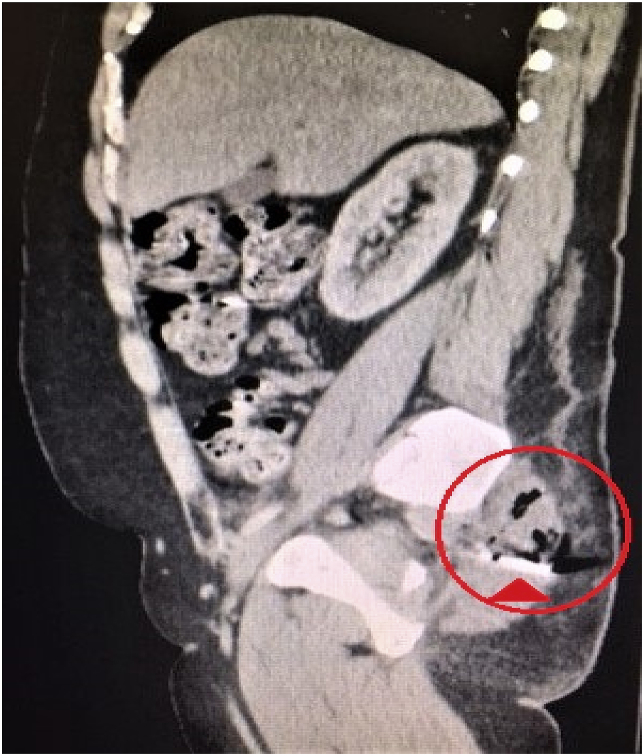
CT sagittal view showing the foreign body screw driver piercing through pelvic muscles with subcutaneous emphysema, circled in red with the arrow head showing the metallic end of the screwdriver.

**Fig. 4:**
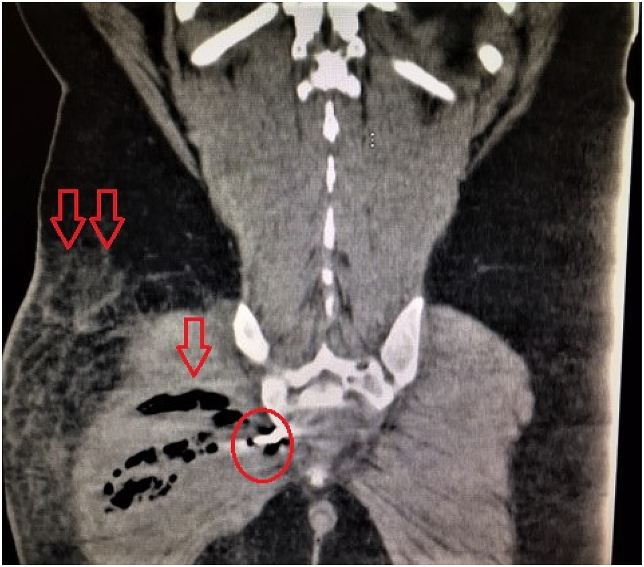
CT coronal view showing the screwdriver end (circled in red) piercing through gluteus muscle with intramuscular (arrow) and subcutaneous emphysema (double arrows).

**Fig. 5:**
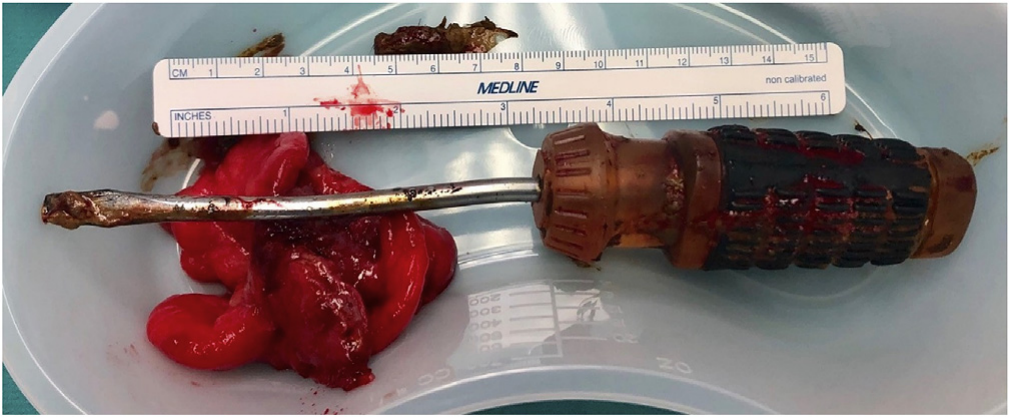
The foreign body screwdriver removed measuring 21 × 3cm along with excised perforated rectum.

A 46-year-old male psychiatric patient presented to the emergency department in septic shock and diffuse abdominal pain from a suspected intra-abdominal source. His past medical history revealed schizophrenia, bipolar disorder, poor compliance with medications, poly-substance abuse, and chronic back pain on outpatient opioid therapy. Symptoms started about 1 week prior to arrival. The septic shock manifest with tachycardia and hypotension. His abdomen was diffusely tender without distension. On digital rectal exam no masses, objects, or blood was appreciated. His white blood cell count was found to be 27,000/μL. CT scan of the abdomen and pelvis revealed a rectal perforation with pneumoperitoneum and the presence of air in the perineum and right buttock tissues [Fig. 1, Fig. 2, Fig. 3, Fig. 4]. He had a foreign object, consistent with a screwdriver in his rectum. Fluid resuscitation was started along with broad-spectrum antibiotics and taken to the operating room. He underwent attempted digital rectal and proctoscopic removal of the foreign object but it was unsuccessful. Blood was encountered in the rectal vault with extensive hard feces during proctoscopic examination. An exploratory laparotomy was then performed which revealed that the sharp metallic shank end of the screwdriver had perforated the rectosigmoid junction and entered the right perineum and buttock. Resection of the perforated rectum with removal of the screwdriver, was performed [Fig. 5]. Due to the septic shock state of the patient, as he required vasopressor medication support and ongoing volume resuscitation during the case, and also had extensive fecal contamination of the abdomen, he was left in intestinal discontinuity and a abdominal wound vacuum dressing was placed. An incision and drainage of a large right buttock abscess was also performed. The patient had ongoing resuscitation in the SICU. He stabilized and was taken back 2 days later for a planned second re-laparotomy, and a proximal end diverting colostomy was performed. The right buttock wound had further necrotizing soft tissue infection was again debrided and another vacuum wound dressing was placed in this cavity.

Our case is unique as intraoperatively we identified a perforation at the rectosigmoid junction from the working end of the screwdriver. This lead to a necrotizing right gluteal infection with subsequent surgeries for debridement and irrigation. The patient recovered and was discharged to Behavioral Health for inpatient treatment of his psychiatric disorders. At 2 weeks follow up the patient was doing well with a functioning ostomy and ostomy reversal was planned.

## 3 Discussion

Despite the numerous reports of anal FB trauma there are no cases in the literature documenting a unique incident such as ours in a psychiatric patient with a screwdriver being inserted through the anus causing a rectal perforation and subsequent pelvic necrotizing soft tissue infection.

In one of the largest single institution series of retained anorectal FBs, Lake et al. [5] found that objects larger than 10 cm, retained longer than 2 days, and those located in the proximal rectum are most likely to require surgical intervention. Our patient required emergent surgery for the bowel perforation and soft tissue infection, however our case supports these findings as the screwdriver measured about 20 cm, was retained for 1 week, and was located at the recto-sigmoid junction.

It is widely agreed upon that when dealing with anorectal foreign bodies the general management should start with more conservative, less invasive approaches such as manual or endoscopic extraction. However, when unsuccessful or when associated with significant injury, as in our case with rectal perforation, a diagnostic laparoscopy or laparotomy is indicated. The surgeon should attempt to “milk” the foreign object distally by applying external lower abdominal pressure however if this is also unsuccessful, the management of this dilemma has evolved with the addition of laparoscopic, endoscopic, and minimally invasive surgical options. A combined approach with the object being pushed down from above and then extracted endoscopically has been described as well. If these measures do not work then extraction via a controlled colotomy is indicated, either laparoscopically or at laparotomy in order to extract the object, which can be followed by primary repair if the patient has limited injury [3,[5], [6], [7]].

The dissent in surgical opinion arises when discussing the surgical management for rectal perforation caused by a FB. The options include primary repair, resection and anastomosis with or without a loop colostomy, or an end diverting colostomy. It is generally agreed upon that for simple colon injuries with lacerations less than 50% circumference involvement, then primary repair should be attempted. However, as the size and gross contamination or spillage of fecal content increases the less unanimity exists among surgeons [2,3,[6], [7], [8]].

Some have used the literature in rectal trauma as a surrogate for anorectal FB. Eshraghi et al. [8] conducted a survey that asked members of the American Association for the Surgery of Trauma (AAST) regarding their preferred management of certain anorectal wounds (not associated with FB) among three options: diverting colostomy (DC), primary repair (PR), or resection and anastomosis (RA). Three hundred twenty-nine of the 449 surgeons returned the completed survey. The investigators concluded that greater than 80% of surgeons agreed on primary repair for simple injuries including perforation with minimal fecal soiling and laceration less than 50% diameter. However as the size and extent of injury increased the majority opinion decreased with 55% of surgeons selecting resection and anastomosis for transected bowel and 41% for blunt rupture. For high velocity gunshot wounds the majority at 56% selected diverting colostomy. This report highlights the necessity for level one evidence and research to provide surgeons with evidence-based standardized approaches for dealing with this unique situation and to ensure the best patient outcomes.

In a landmark prospective randomized trial with 268 patients with anorectal injury, without FB, researchers compared colostomy versus primary repair. Stone and Fabian [9] concluded that primary repair was preferred to colostomy in a select group of patients. However a cohort of patients deemed high risk for failure of repair were excluded. The exclusion criteria included shock, blood loss greater than 20%, more than two intra-abdominal organ system injuries, and gross fecal contamination.

Since this landmark trial there have been 7 additional studies on anorectal trauma without FB, that compared colostomy versus primary repair and those patients deemed high risk for failure of repair were not excluded from the randomization process. All of the studies found promising results in favor of primary repair regardless of the mentioned risk factors [[9], [10], [11], [12], [13], [14], [15]].

The most recent multicenter prospective trial sponsored by the Committee on Multicenter Clinical Trials of the AAST evaluated the safety of primary anastomosis versus diversion and the development of colon-related abdominal complications. The study included 297 patients with 197 patients (66.3%) managed by primary anastomosis and 100 (33.7%) by diversion. Demetriades et al. [16] found no statistically significant difference in outcome when comparing primary anastomosis with diversion using multivariate analysis adjusting for three identified independent risk factors (severe fecal contamination, transfusion of ≥4 units of blood within the first 24 hours, and single-agent antibiotic prophylaxis) or the risk factors previously mentioned. However, the investigators acknowledged possible weaknesses of the study including not investigating factors such as severe bowel edema or bowel ischemia.

In our case due to his critical clinical status of septic shock requiring resuscitative fluids and high dose vasopressor support, with extensive fecal contamination, and necrotizing soft tissue infection, we elected for a more conservative and expeditious operation encompassing resection and end colostomy. The option of resection and primary anastomosis would have carried significant risk as vasopressors are known to increase anastomotic leaks due to bowel ischemia [17].

## 4 Conclusion

We present a rare case of a rectal foreign body screwdriver causing bowel perforation and soft tissue infection that required operative intervention. Our case was unique as to the delayed presentation of the patient from insertion of the foreign body and seeking medical attention due to his psychiatric disease. Intraoperatively we identified a perforation at the rectosigmoid junction where the shank and head of the screwdriver were identified to have had perforated into the right lateral pelvic wall. This injury lead to a necrotizing right gluteal infection with subsequent surgeries for debridement and irrigation. This case highlights the importance of maintaining a high index of suspicion when encountering psychiatric disease patients with nonspecific lower abdominal or anorectal pain in patients with inconsistent history and presentation. For anorectal foreign bodies causing perforation with contamination controversy exists regarding the type of surgical treatment. We elected for a more patient centered approach that takes into account the patient's overall clinical status, degree of injury and contamination, and the amount of time the object has been retained. More research is needed on the surgical outcomes of resection and primary anastomosis versus traditional methods. Specifically patients displaying hemodynamic lability and requiring high vasopressor support intraoperatively, following foreign body anorectal injury to ensure optimal patient outcomes.

## 5 Learning points

• Clinicians must maintain a high index of suspicion when encountering patients with nonspecific lower abdominal or anorectal pain in patients with inconsistent presentations, especially those with psychiatric disease or a history of self-harm.
• For anorectal foreign bodies the general management should start with more conservative less invasive approaches such as manual or endoscopic extraction or a combination of the two.
• When unsuccessful or when associated with significant injury, as in our case with rectal perforation, a diagnostic laparoscopy or laparotomy is indicated.
• The surgeon should attempt to “milk” the foreign object distally by applying external lower abdominal pressure in an effort to push the foreign body distally and aid in transrectal extraction. However if this is also unsuccessful, the management of this dilemma has evolved with the addition of laparoscopic, endoscopic, and minimally invasive surgical options.
• If these measures do not work then extraction via a controlled colotomy is indicated, either laparoscopically or at laparotomy in order to extract the object which can be followed by primary repair if the patient has limited injury
• Controversy exists regarding the surgical management for rectal perforation caused by a FB. The options include primary repair, resection and anastomosis, or diverting colostomy.
• As the size of the perforation and gross contamination or spillage of fecal material increases there is less unanimity among surgeons as to the exact surgical approach.

### Provenance and peer review

Not commissioned, externally peer reviewed.

## Ethical Approval

This case report was conducted in compliance with ethical standards. Informed written consent has been obtained and all identifying information is omitted.

## Sources of funding

None.

## Author contribution

Youssef Shaban, Adel Elkbuli, Vasiliy Ovakimyan, Dessy Boneva, Mark McKenney, Shaikh Hai– Conception of study, acquisition of data, analysis and interpretation of data.

Youssef Shaban, Adel Elkbuli, Vasiliy Ovakimyan, Rachel Wobing, Dessy Boneva, Shaikh Hai, Mark McKenney - Drafting the article.

Shaikh Hai, Mark McKenney – Management of case.

Youssef Shaban, Adel Elkbuli, Vasiliy Ovakimyan, Rachel Wobing, Dessy Boneva, Shaikh Hai, Mark McKenney – Critical revision of article and final approval of the version to be submitted.

## Trial registry number – ISRCTN

NA.

## Guarantor

Shaikh Hai.

Mark McKenney.

## Research registration unique identifying number (UIN)

This is a case report study.

## Declaration of competing interest

None.
